# Validating Predictors of Disease Progression in a Large Cohort of Primary-Progressive Multiple Sclerosis Based on a Systematic Literature Review

**DOI:** 10.1371/journal.pone.0092761

**Published:** 2014-03-20

**Authors:** Jan-Patrick Stellmann, Anneke Neuhaus, Christian Lederer, Martin Daumer, Christoph Heesen

**Affiliations:** 1 Institute for Neuroimmunology and Clinical MS Research (inims), University Medical Center Hamburg-Eppendorf, Hamburg, Germany; 2 Sylvia Lawry Centre for Multiple Sclerosis Research, Munich, Germany; 3 Trium Analysis Online GmbH, Munich, Germany; 4 Department of Neurology, University Medical Center Hamburg-Eppendorf, Hamburg, Germany; Institute Biomedical Research August Pi Sunyer (IDIBAPS) - Hospital Clinic of Barcelona, Spain

## Abstract

**Background:**

New agents with neuroprotective or neuroregenerative potential might be explored in primary-progressive Multiple Sclerosis (PPMS) - the MS disease course with leading neurodegenerative pathology. Identification of patients with a high short-term risk for progression may minimize study duration and sample size. Cohort studies reported several variables as predictors of EDSS disability progression but findings were partially contradictory.

**Objective:**

To analyse the impact of published predictors on EDSS disease progression in a large cohort of PPMS patients.

**Methods:**

A systematic literature research was performed to identify predictors for disease progression in PPMS. Individual case data from the Sylvia Lawry Centre (SLC) and the Hamburg MS patient database (HAPIMS) was pooled for a retrospective validation of these predictors on the annualized EDSS change.

**Results:**

The systematic literature analysis revealed heterogeneous data from 3 prospective and 5 retrospective natural history cohort studies. Age at onset, gender, type of first symptoms and early EDSS changes were available for validation. Our pooled cohort of 597 PPMS patients (54% female) had a mean follow-up of 4.4 years and mean change of EDSS of 0.35 per year based on 2503 EDSS assessments. There was no significant association between the investigated variables and the EDSS-change.

**Conclusion:**

None of the analysed variables were predictive for the disease progression measured by the annualized EDSS change. Whether PPMS is still unpredictable or our results may be due to limitations of cohort assessments or selection of predictors cannot be answered. Large systematic prospective studies with new endpoints are needed.

## Introduction

Primary-progressive Multiple Sclerosis (PPMS) might be understood as the MS disease course with the lowest inflammatory and highest neurodegenerative impact on disability.[1], [2] The clinical disease course rarely shows relapses but a chronic progressive accumulation of disability. MRI markers of inflammation as new T2-lesions or gadolinium enhancing lesions are less frequent changing than for relapsing-remitting Multiple Sclerosis (RRMS).[1], [3]–[5] Similar to RRMS, the disease course of PPMS is highly variable. While 25% of the patients need a walking aid after 7.3 years, another 25% remain almost fully ambulatory after 25 years.[5]

While a number of different anti-inflammatory treatments for RRMS are already available or will be approved in the next years,[2] several placebos controlled randomized trials of anti-inflammatory drugs in PPMS failed to reduce the risk for disability progression over 2–3 years.[6]–[11] These negative results may be due to a lack of understanding of the natural history of PPMS and low inflammatory activity of PPMS or insensitive endpoints leading to insufficient study designs. So far, a progression in disability measured by the Expanded Disability Status Scale (EDSS) and confirmed after 3 or 6 months has been used in phase II and III studies as primary endpoint.[6]–[11] This endpoint definition is problematic as the EDSS suffers from a high inter- and intra-rater variability.[12] Further on, “sustained changes of EDSS” over a period of 3 to 6 months cannot be used as a surrogate of unremitting disability and EDSS landmarks of disability as like the need of a walking aid occur only rarely in clinical studies.[12] In addition, EDSS seems not sensitive enough to be endpoint for short term phase II trials with a duration of 6 to 12 months, which would be needed to explore new drugs. The International Collaborative on Progressive MS recently published a research agenda for progressive MS, which claimed new outcome parameters and new phase II clinical trial strategies as 2 out of 5 major research needs.[2] Similar demands are published by the European Medicines Agency (EMA) concerning future clinical MS research.[13] As long as alternative and reliable surrogate markers for long term progression in MS are missing, one option of reducing sample sizes, gaining power and allowing shorter trials may be the inclusion of patients with a fast progression within 12 to 36 months. This is important since PPMS may be seen as the best patient group for future trials on neuroprotective or neuroregenerative agents as fluctuation in clinical presentation and since disabilities due to relapses are rare.

Assessing predictors of disability progression in PPMS may therefore be a feasible approach to identify patients with high short-term risk of disease progression. The outcome of a retrospective analysis of 552 PPMS patients from the British Columbia MS database was that a younger age at disease manifestation as well as sensitive symptoms at onset were associated with a longer time to EDSS 6.0.[1] The median time to EDSS 6.0 was 14 years and substantially longer then reported from the Lyon (n = 282) and London Ontario (n = 219) cohorts (7.1 respectively 8.0 years). These differences may be based on different attrition rates. Gender as well as superimposed relapses were not associated with time to EDSS 6.0 in these three databases.[1], [14], [15] In contrast, a prospective European study (n = 101) found a faster progression in male patients, in subjects with a shorter disease duration at baseline or a slower 10 m Timed Walk Test (TWT).[16] Further on, EDSS progression and MRI brain atrophy rates over the first 2 years were predictive for the EDSS after 10 years. In a combined analysis, TWT and EDSS progression over the first 2 years were the best predictors for the EDSS after 10 years.[16] A good predictive value of early changes in EDSS and Timed 25 Foot Walk (T25FW) for long term EDSS deterioration in a cohort of 181 PPMS was again published recently.[17] In summary, most of these results are heterogeneous and lack confirmation.

As long as more prospective and validated data is missing, combination of retrospective individual case data might contribute to the understanding of the natural disease course of PPMS, predictors of disease progression and the design of future treatment trials in these patients. Based on this idea, case data from the Sylvia Lawry Centre (SLC) and the Hamburg MS patient database (HAPIMS) were analysed.

## Methods

This study was designed to validate predictors of disease progression in PPMS which are already published. Predictors were identified by systematic literature research. Due to this strict methodical approach, an explorative analysis of other possible predictive variables was not included.

### Systematic literature search

A comprehensive literature research within the Pubmed database was performed (last access in January 2013) by one reviewer (JPS) with the following keywords: ‘natural history progressive multiple sclerosis', ‘predictors of disease progression multiple sclerosis’ and ‘predictors of disability in “progressive multiple sclerosis”'. After removing duplicates (n = 21), the results of the electronic search (n = 219) were screened by headers and abstracts. One further article based on cross-references was included. Only full-length original English journal publications were reviewed to identify studies that met the following a priori defined criteria (n = 13): (1) data from at least 40 PPMS Patients without other MS disease courses: This sample sizes has a 90% power to detect a significant correlation with R^2^ = 0.5 and p<0.05 in linear models. (2) a follow-up of at least 3 years, (3) not only MRI predictors reported and (4) published between 1980 and January 2013. Record selection, exclusion and inclusion of studies according to the PRISMA guidelines is presented in Figure S1.[18] JPS and CH performed exclusions. From each study we extracted whether a predictor was investigated and if there was a significant association with the outcome. Based on this research and the available patient data in the pooled cohort we chose predictors for validation. As primary outcome we used the annualized change of EDSS (delta-EDSS). All steps of this validation plan were predefined in a statistical analysis plan (see below).

### Case dataset and Ethics Statement

For this study the individual case database from the Sylvia Lawry Centre (SLC) and the University of Hamburg MS outpatient clinic (HH) was accessed. As all data was anonymised and collected approved by IRB (neither an additional approval for this retrospective study was needed nor was it specifically waived by an IRB). The SLC database consists of anonymised data from various data-donors, of natural history studies and randomized controlled clinical trials. The data donors collected the data with appropriate consent forms. Without explicit permission of the data-donor even his personal data is anonymised. Since 1998 the Hamburg MS outpatient clinic has been collecting patient data along their clinical visits. All patients included in the dataset gave written permission that their clinical data may be used for anonymous medical analyses. Besides general information about disease courses, disease onset and treatment, the data includes at each visit “Expanded Disability Status Scale” (EDSS), “Multiple Sclerosis Functional Composite” (MSFC) and “Quality of life” (QoL) assessments. Until 2011 datasets of more than 3.000 patients with a total of about 10.000 visits have been included into this database.

Both databases were screened for PPMS patients who had at least two assessments. To guarantee the homogeneity of the two datasets, only natural history cohort data from the SLC was included and data from randomized controlled trials excluded. None of the two databases collected natural history cohort MRI data in a regular manner. Walking tests and MSFC data were not available in the SLC dataset and therefore none of these variables were used for analyses. Following baseline variables were extracted: Age, sex, time since first symptoms, time since diagnosis and if first symptoms affected the motor system. For baseline and follow-up visits we extracted EDSS and date of assessments. EDSS scores were not confirmed after 3 or 6 months. Primary outcome for disease progression was the change of EDSS over time. To control for variable visit intervals we calculated the annualized change of EDSS (delta EDSS  =  (EDSS_last_date_ – EDSS_first_date_)/(last_date – first_date)).

### Statistics

Descriptive statistics were performed and non-parametric statistics were used for further analyses, as the EDSS is an ordinal-scale. In addition a linear mixed effects model was used to calculate the average EDSS increase per year. Spearman's rank correlations were calculated for ordinal or metric predictors. The Wilcoxon rank-sum test was chosen for dichotomous predictors (e.g. gender). All analyses were performed for the pooled dataset as well as for each database (SLC or HH) separately. A result was considered statistically significant with a p-value less than 0.05. Models with multiple explanatory variables were planned in case simple analyses revealed more than one significant predictor to estimate relative contribution of each predictor to the outcome or in case the systematic literature analyses revealed a conclusive assumption about associations between predictors. No additional explorative analyses were performed. Open source software “Software R” was used for analyses.[19]

## Results

### Systematic literature research

We identified a total of 3 prospective studies and reports from another 5 retrospective cohorts.[1], [5], [15]–[17], [20]–[26] The available data about predictors of disease progression in PPMS were very heterogeneous and included rarely information about what would allow a statistically meaningful meta-analytic synthesis. For the majority of the predictors only a subset of studies reported results. On a descriptive level, 4 cohorts described an association of age at onset with progression risk.[1], [5], [16], [26] Three cohorts did not.[20], [23], [27] We found a worse prognosis in males in 2 studies while 5 did not find an association between gender and EDSS-progression.[1], [5], [16], [20]–[24] Three publications reported a missing association between first symptoms and time to EDSS-milestones.[5], [20], [23] In one study an increased risk for faster disability progression was associated with motor symptoms at onset and another study reported an increased risk for patients with involvement of more than 2 functional systems at onset.[1], [21] Slowly progressive or more rapid onset of first symptoms did not correlate with disease progression in 2 studies, nor did superimposed relapses (3 publications).[15], [21]–[23], [26] Four studies found an early EDSS change predictive for later EDSS changes, one did not.[16], [17], [21]–[23] A summary of our findings including various variables investigated only in 1 or 2 studies is given in Table S2.

Based on the results of the systematic literature research and the available variables in our data, we generated the following hypotheses for validation.

1. Younger age at onset is associated with slower progression measured by delta-EDSS.
2. Male sex is associated with faster progression measured by delta-EDSS.
3. Motor symptoms at onset are associated with faster disability progression.
4. Delta-EDSS over the first 2 years predicts Delta-EDSS after year 2.

### Patient cohorts

We identified a total of 597 PPMS patients (SLC: n = 302, HH: n = 295) with a total of 2503 EDSS assessments (SLC = 1111, HH = 1392). All descriptive statistics are summarized in table 1. The pooled cohort consists out of 54% females with a mean age of 40.4 years at baseline. Disability increased from baseline mean EDSS 4.3 over a mean follow-up of 4.4 years to a mean EDSS of 5.5 at the last visit. The mean annualized increase of EDSS was 0.35. The predicted average EDSS increase per year based on the linear mixed effects model was 0.24. The cohorts from the two databases were quite similar except the fact that SLC patients had a non-significant higher rate of motor system affection as first symptoms, a significant longer mean follow-up and a non significant slower disease progression measured by mean delta EDSS.

**Table 1:** Descriptive statistics.

	All	HH	SLC
	(n = 597)	(n = 302)	(n = 295)
**Females** n (%)	285 (54%)	121 (52%)	164 (56%)
**Age** mean (sd)	40.4 (11.1)	41.6 (11.2)	39.5 (10.9)
**Motor symptoms as first manifestation** n (%)	179 (49%)	83 (35%)	96 (73%)
**EDSS baseline**			
mean (sd)	4.3 (1.8)	4.1 (1.8)	4.4 (1.8)
median (range)	4.0 (0;9.0)	4.0 (0;9.0)	4.0 (0;9.0)
**Number of EDSS measurements**	2503	1111	1392
**Follow-Up (yrs)** mean (sd)	4.4 (4.3)	3.7 (3.1)	4.9 (4.9)
**last EDSS** mean (sd)	5.5 (1.9)	5.0 (1.8)	5.8 (2.0)
**delta EDSS** mean (sd)	0.35 (1.51)	0.41 (2.15)	0.30 (0.65)

Descriptive statistics for the pooled dataset and separately for the datasets from Hamburg (HH) respectively from the Sylvia Lawry Centre (SLC).

**Figure 1:**
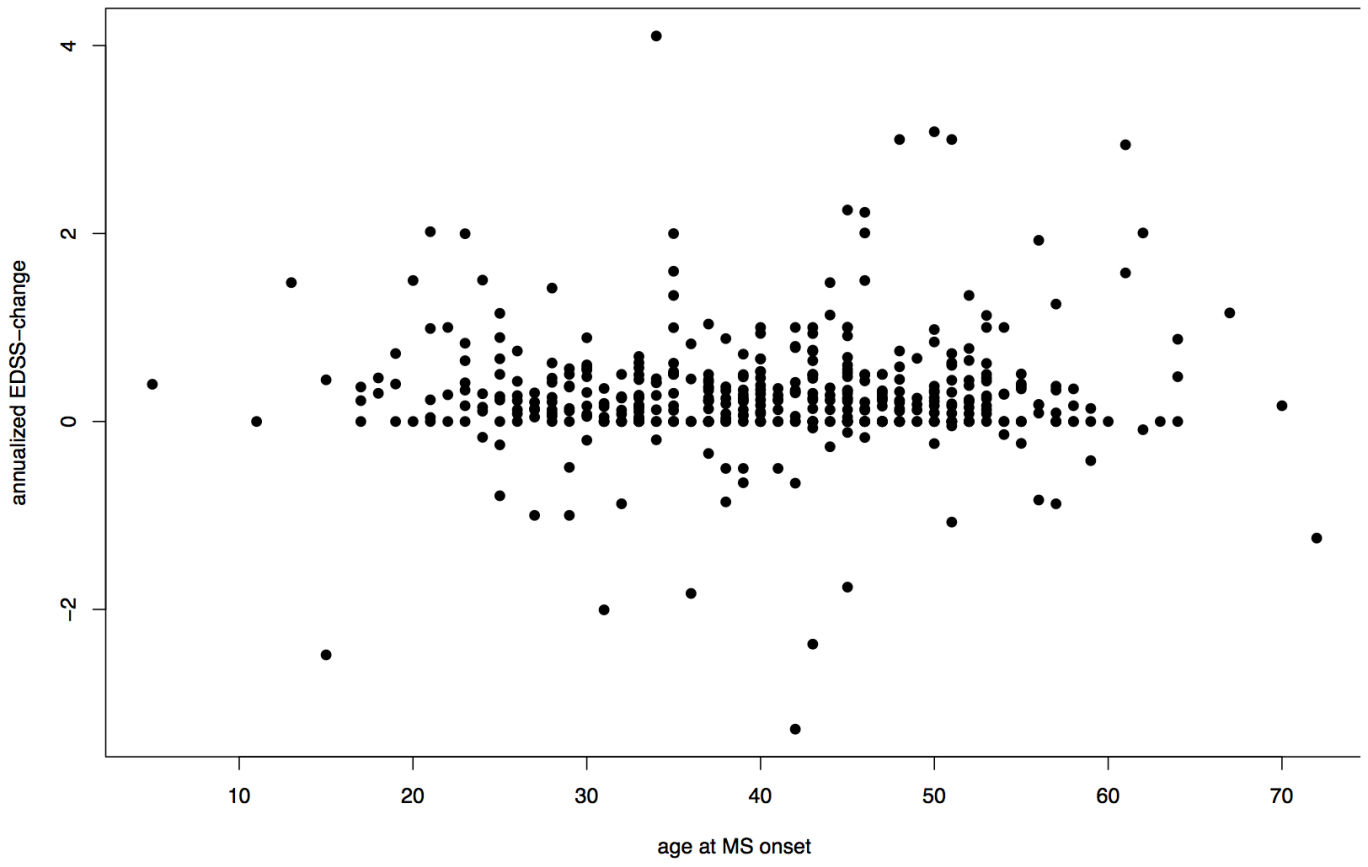
Association between Age at Onset and annualized EDSS progression. delta-EDSS = annualized difference between first and last EDSS assessment, Spearman's rank correlation r = −0.0359, p = 0.4508. One outlier with an annualized delta-EDSS >10 was excluded.

Age at onset was not correlated with delta-EDSS in the pooled cohort (Spearman's rank correlation  =  −0.0359, p = 0.4508, Figure 1) nor in each cohort (SLC p = 0.4752, HH p = 0.0693).

The difference of median delta-EDSS between females (0.1710) and males (0.2361) was not significant in the pooled cohort (p = 0.1996, Figure 2). This finding was the same in a separate analysis of the HH and SLC cohort. Groups split by absence or presence of motor symptoms at disease onset did not impact on in their delta-EDSS (p = 0.2418, Figure 3) in the whole cohort as well as separated analysis of Hamburg and SLC data. There was no significant correlation between delta-EDSS in the first two years after baseline and delta-EDSS from the third year on (Spearman's rank correlation  =  −0.0445, p = 0.6536, Figure 4). In contrast to the other analyses, separate analyses for each cohort revealed a discrepancy between the cohorts. While there was no significant correlation between early and late delta-EDSS within the SLC cohort (Spearman's rank correlation  =  0.3873, p = 0.0506), we found a significant inverse correlation (Spearman's rank correlation  =  −0.2253, p =  0.0474) in the HH cohort.

**Figure 2:**
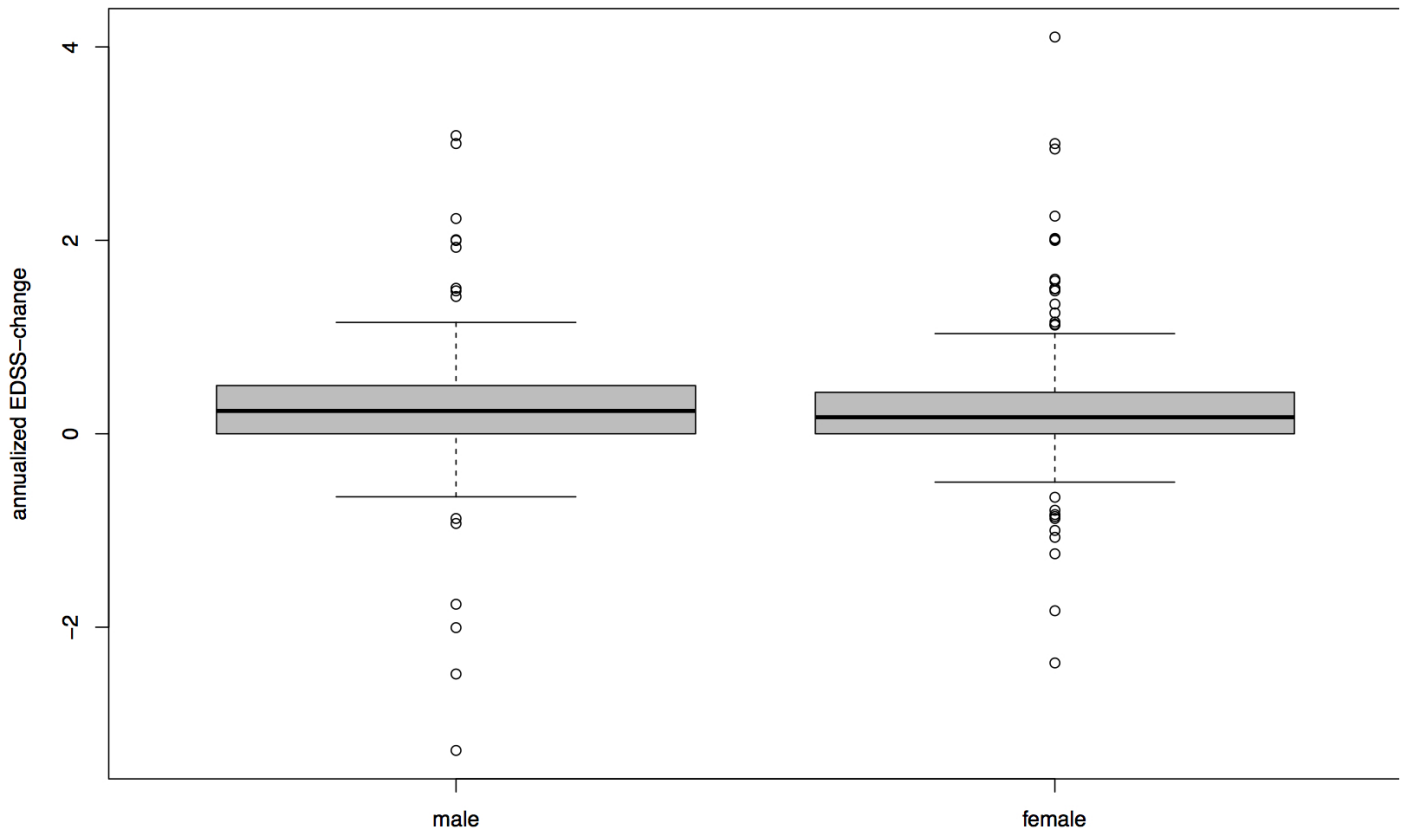
Median delta-EDSS in Women and Men. Boxplot with median, quartiles, 95% interval whiskers and outliers, delta-EDSS = annualized difference between first and last EDSS assessment, Wilcoxon rank-sum test: p = 0.1996. Two outliers with an annualized delta-EDSS >10 were excluded.

**Figure 3:**
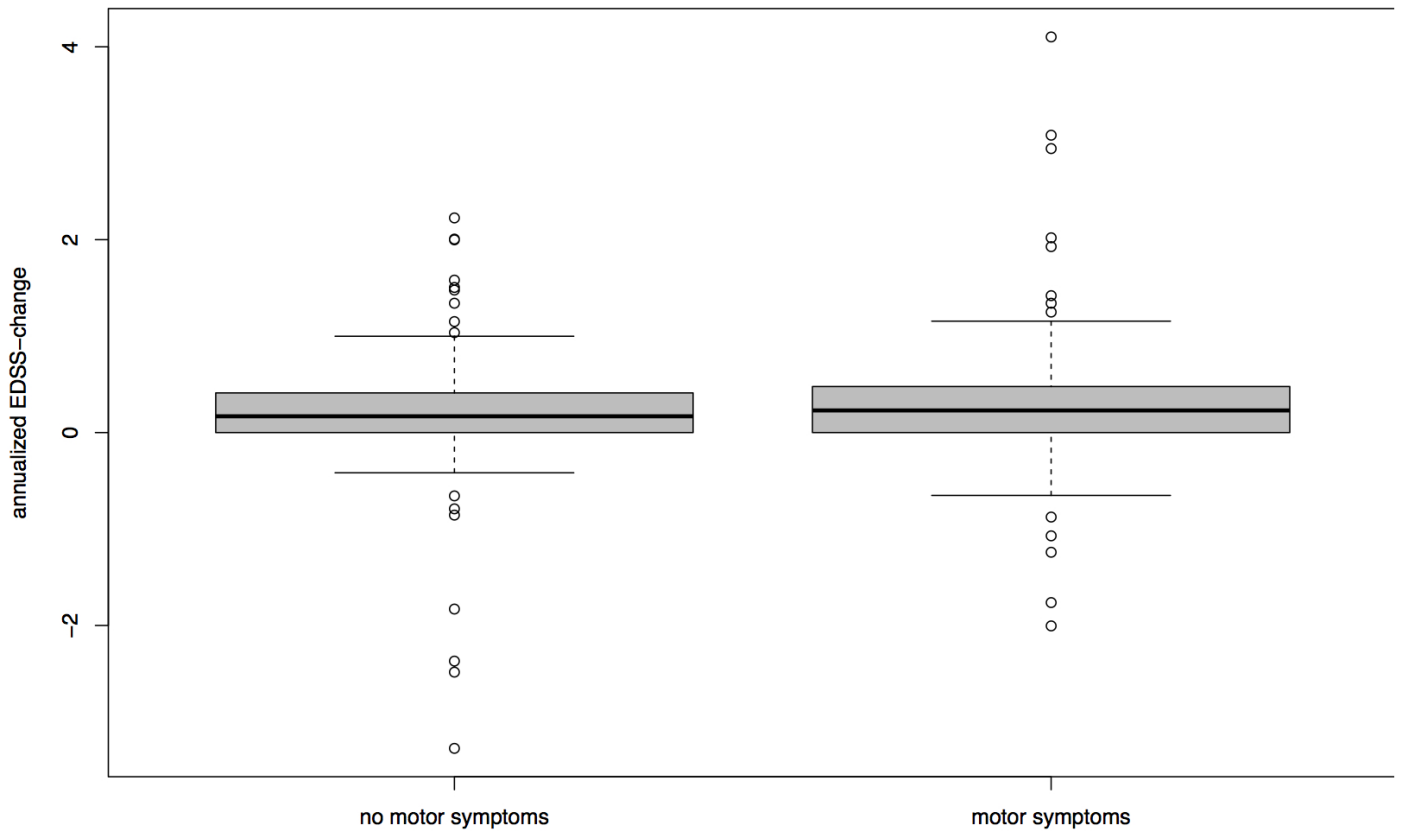
Median delta-EDSS and Type of First Symptoms. Groups split by absence or presence of motor symptoms at disease onset. Boxplot with median, quartiles and 95% interval whiskers, delta-EDSS = annualized difference between first and last EDSS assessment, Wilcoxon rank-sum test: p = 0.2418.

**Figure 4:**
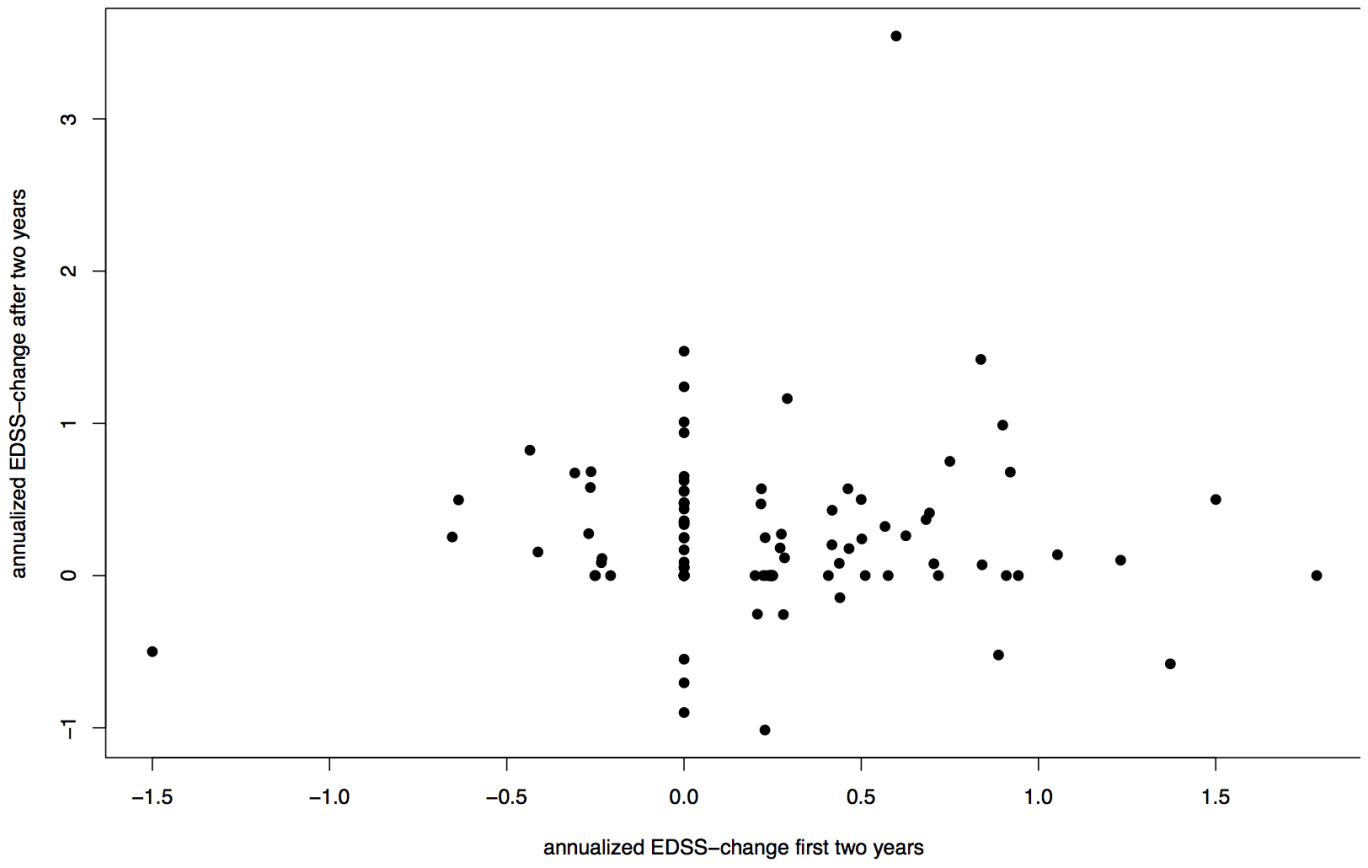
Association between Early and Late EDSS-Progression. Delta-EDSS in the first two years after baseline and delta-EDSS from year three on, delta-EDSS = annualized difference between first and last EDSS assessment, Spearman's rank correlation  =  −0.0445, p = 0.6536.

## Discussion

To the best of our knowledge, this is the yet largest analysis of patient-level pooled natural history cohort data in PPMS. Baseline characteristics showed a typical PPMS population with a nearly equal gender ratio and a mean age above 40 years. We could not confirm gender, age of onset, type of first symptoms or early EDSS-progression as predictors of the long-term EDSS dynamic in primary progressive MS. Explorative analyses of other variables were not performed as we followed a strict validation policy. If these findings represent a true unpredictable disease course in PPMS or point more to the insensitivity of the EDSS as outcome in progressive MS remains unclear. The lack of formally statistically significant associations of possible predictors and disability in our study does not finally exclude an association but taken together with the heterogeneous results from other studies a strong association seems very unlikely. Only for the Hamburg cohort we found a significant, although weak association between early and late EDSS-progression, which might indicate a slower disease progression in the later disease course after initially fast progression and vice versa. As the analysis of the SLC cohort showed a trend towards an inverse correlation and R2 values were low, we interpret these divergent findings together as failed validation of our predefined hypothesis that delta-EDSS is a predictor for further progression.

The investigated variables were selected for validation from previous publications including large natural history cohorts and 3 prospective cohort studies.[1], [14]–[16], [24] These studies show a high variability of findings: The only variable that showed a consisting finding in at least three publications was superimposed relapses which were not associated with disease progression.

In contrast to our study, all studies were designed to explore the available data but did not include a validation strategy. Only one publication mentions a failed confirmation of a previously generated hypothesis about the worse prognosis of multiple functional systems affected at disease onset.[27] Due to the already existing heterogeneity of findings, we decided against an additional explorative study and chose a validation strategy. Based on these findings, it is not possible to give any recommendations about inclusion criteria for treatment trials in progressive MS nor does the available data allow an individualized risk counselling.

All previous studies as well as our own work used EDSS related endpoints. The predicted annual average EDSS increase of 0.24 in our cohort was similar to previous analyses in secondary progressive MS (2-year increase: 0.50±0.92).[28] Two major limitations of the EDSS may explain the unsuccessful attempts to identify predictors of disease progression in progressive MS. First, a very high inter- and intra-rater variability and the low risk for reaching one of the disability landmarks in the short-term restrict its use in short prospective cohorts or even treatment trials.[12], [29] To our knowledge sustained EDSS-progression as an outcome in PPMS trials and its association with the long-term risk of disability has not been investigated in detail. But EDSS-progression failed to predict the long-term risk of disability accumulation in other MS disease courses.[29] This point of view has also been risen by the International MS Collaborative Consortium and legal authorities.[2], [13], [30] Second, EDSS landmarks are mainly defined by mobility restrictions ignoring other functional systems that may have a major impact on disability. The knowledge about the accumulation of disability in different functional systems is rare. In fact, most studies from our systematic literature research report symptoms only at disease onset, which mainly includes weakness of lower limbs and ataxia, vision impairment in up to 41% and cognitive impairment in up to 6%.[5], [20], [27], [31] Interestingly, in a Brazilian cohort cognitive impairment was found in 73% of PPMS patients compared to 46% in a RRMS control group.[32] Other data show a decrease in cognitive function of PPMS patients through time.[33], [34] These conflicting findings show that much more work is needed to understand the clinical impact of PPMS.

The heterogeneity of the previous published studies as well as our findings may rather reflect the limitations of the selected outcomes than the unpredictability of PPMS. Objective new outcomes reflecting real-life implications of MS patients are needed as stated by the The International Collaborative on Progressive MS.[2] Even a simple walking test like the Timed 25 foot walk (T25FW) shows a better predictive value for patient reported impairment than the EDSS.[35] Post-hoc analyses of the Rituximab in PPMS trial showed a significant effect of the treatment over placebo if a composite outcome of EDSS and T25FW was used.[11], [36] We suggest step-by-step evaluation of reliable outcomes e.g. for mobility, cognitive impairment or visual function. Besides the methodical considerations, quality of life research support focussing these three functional systems as key value domains from patients' perspective. [37]

Limitations of our and other natural history cohort studies are their retrospective design and the lack of a standardized follow-up. Patients with a more rapid increase of disability might have lost the ability to visit the involved MS centres. Further on, missing treatment options for several MS related impairments as fatigue or vision loss might lead to a selection bias towards subjects with treatable symptoms as spasticity or pain. This might bias representativeness of our findings for the natural disease course in primary progressive MS. Our results are limited as the available data in our cohorts did not allow to include walking tests, neuropsychology, sustained EDSS-progression or MRI parameters in our analyses. Some hypotheses, as the predictive value of gait tests generated the European 10 year prospective cohort could not be validated.[16] Future studies need to address prevalence of symptoms at disease onset and evolution through time in a prospective manner including validation strategies and development of new outcomes.

## Conclusion

Our study could not validate gender, age at onset, type of first symptoms or early EDSS-progression as predictive for the EDSS dynamic in PPMS. Prospective cohort data of PPMS are yet very limited. Besides larger cohorts, new outcome measures for progressive MS are needed, as the EDSS seems to be too imprecise to detect disease progression in these patients.

## Supporting Information

Figure S1 **PRISMA Flow Chart.**
(EPS)

Table S1 **PRISMA checklist.**
(DOCX)

Table S2 **Overview – Published predictors of disease progression in PPMS.** PPMS  =  primary progressive MS, SPMS  =  secondary progressive MS, +  =  positively associated with disease progression, -  =  negatively associated with disease progression, 0  =  no association, *direction of association either positively or negatively not reported, Br  =  EDSS FS Brainstem, NP  =  not published, GNDS  =  Guy's Neurological Disability Scale, NHC = natural history cohort data, PC = prospective cohort, §  =  the previous published 5 year data (36) are not shown, MRI results are not included.
(DOCX)
