# Supplementary material for: Validating Predictors of Disease Progression in a Large Cohort of Primary-Progressive Multiple Sclerosis Based on a Systematic Literature Review
Source: PLoS One. 2014 Mar 20;9(3):e92761. doi: 10.1371/journal.pone.0092761 (PMC3961431; doi:10.1371/journal.pone.0092761)
Supplement: Table S2 — Overview – Published predictors of disease progression in PPMS. PPMS = primary progressive MS, SPMS = secondary progressive MS, + = positively associated with disease progression, - = negatively associated with disease progression, 0 = no association, *direction of association either positively or negatively not reported, Br = EDSS FS Brainstem, NP = not published, GNDS = Guy's Neurological Disability Scale, NHC = natural history cohort data, PC = prospective cohort, § = the previous published 5 year data (36) are not shown, MRI results are not included. (DOCX) [file pone.0092761.s003.docx]

|  | **Publication** | **Andersson 1999 [20]** | **Cottrell 1999 [21,27]** | **Confavreux 2000 + 2003 [14,22,23]** | **Tremlett 2005 [5]** | **Rovaris 2006 [24]** | **Kremenchutzky 2006 [15]** | **Khaleeli 2008 [16]§** | **Koch 2009 [1]** | **Khaleeli 2010 [25]** | **Bosma 2012 [17]** | **Calabrese 2012 [26]** |
| --- | --- | --- | --- | --- | --- | --- | --- | --- | --- | --- | --- | --- |
|  | **Country** | San Francisco, USA | London Ontario, Canada | Lyon, France | British Columbia, Canada | Italy | London Ontario, Canada | Europe | British Columbia, Canada | London, UK | Amsterdam, Netherlands | Italy |
|  | **Design** | NHC, all patients at centre 1993-96, telephone follow | NHC, all patients at centre 1979-84, clinical or telephone follow-up | NHC, all patients at centre 1976-97, clinical follow-up | NHC, all patients with a clinical follow-up > 5 years and disease duration > 15 years | PC, selected patients with PPMS that gave informed consent | NHC, all patients with a clinical follow-up > 5 years and disease duration > 15 years | PC, selected patients with PPMS that gave informed consent | NHC, all patients with a clinical follow-up > 5 years and disease duration > 15 years | PC, selected patients with PPMS that gave informed consentProspective cohort | NHC, PPMS patients from a health status programme | PC, recruited consecutively from 2005 to 2006 |
|  | **n** | 83 | 216 | 282 | 352 | 54 | 359 | 101 | 424 | 45 | 181 | 47 |
|  | **Follow-up (years)** | NP | Mean 23 | NP | mean 17.2 | median 4.8 | Mean 25 | 10 | Median 13.1 | 5 | Mean 7.5 | 5 |
|  | **Outcome** | Time to EDSS 6, 6.5 and 7 | Time to DSS 6,8 and 10 | Time to DSS 4,6 and 7 | Time to EDSS 6 and 8 | EDSS progression over study period | Time to DSS 6,8 and 10 | EDSS progression at year 10 | Time to EDSS 6 and 8 | EDSS (ordinal logistic) | EDSS (ordinal logistic) | EDSS (ordinal logistic) |
| **Disease characteristics** | **Gender** | 0 | 0 - except DSS10 earlier in males | Male have higher risk | 0 | 0 | NP | Male have higher risk | 0 | NP | NP | NP |
|  | **Age at onset** | 0 | 0 | 0 | - | NP | NP | NP | - | - | NP | associated* |
|  | **First symptoms** | 0 | > 3 FS involved worse | 0 | 0 | NP | NP | NP | No motor system = slower progression | NP | NP | NP |
|  | **Progressive or sub acute onset** | NP | 0 | NP | NP | NP | 0 | NP | NP | NP | NP | NP |
|  | **Superimposed relapses** | NP | NP | 0 | NP | NP | 0 | NP | NP | NP | NP | 0 |
| **Baseline** | **Disease duration** | NP | NP | NP | NP | 0 | NP | + | NP | NP | NP | associated* |
|  | **EDSS** | NP | NP | NP | NP | - | NP | 0 | NP | NP | NP | NP |
|  | **short walking tests (10 m walk, T25FW)** | NP | NP | NP | NP | NP | NP | + | NP | NP | NP | NP |
|  | **9HPT** | NP | NP | NP | NP | NP | NP | 0 | NP | NP | NP | NP |
| **Baseline MRI** | **GD-enhancement** | NP | NP | NP | NP | NP | NP | NP | NP | 0 | NP | 0 |
|  | **T2 Lesion Load** | NP | NP | NP | NP | NP | NP | NP | NP | + | NP | 0 |
|  | **Grey Matter Fraction** | NP | NP | NP | NP | NP | NP | NP | NP | NP | NP | associated* |
|  | **Cortical Lesion Volume** | NP | NP | NP | NP | NP | NP | NP | NP | NP | NP | associated* |
| **Early changes** | **EDSS progression** | NP | Time to DSS 3 predicts time to DSS 8 | 0 | Time to EDSS 6 predicts time to EDSS 8 | NP | NP | + | NP | NP | + | NP |
|  | **T25FW** | NP | NP | NP | NP | NP | NP | NP | NP | NP | + | NP |
|  | **9HPT** | NP | NP | NP | NP | NP | NP | NP | NP | NP | 0 | NP |
|  | **GNDS** | NP | NP | NP | NP | NP | NP | NP | NP | NP | 0 | NP |
|  | **T2 Lesion Load** | NP | NP | NP | NP | NP | NP | NP | NP | NP | NP | 0 |
|  | **Cortical Lesions** | NP | NP | NP | NP | NP | NP | NP | NP | NP | NP | 0 |
